# Evidence for Attentional Phenotypes in Infancy and Their Role in Visual Cognitive Performance

**DOI:** 10.3390/brainsci10090605

**Published:** 2020-09-03

**Authors:** Shannon Ross-Sheehy, Esther Reynolds, Bret Eschman

**Affiliations:** 1 Department of Psychology, University of Tennessee, Knoxville, TN 37996, USA; ereyno14@utk.edu; 2 Department of Psychology, Florida International University, Miami, FL 33199, USA; beschman@fiu.edu

**Keywords:** infant attention, visual orienting, saccades, infant development, cognitive development, visual attention, saccadic reaction time, eye-tracking, visual working memory

## Abstract

Infant visual attention rapidly develops during the first year of life, playing a pivotal role in the way infants process, learn, and respond to their visual world. It is possible that individual differences in eye movement patterns shape early experience and thus subsequent cognitive development. If this is the case, then it may be possible to identify sub-optimal attentional behaviors in infancy, before the emergence of cognitive deficit. In Experiment 1, a latent profile analysis was conducted on scores derived from the Infant Orienting with Attention (IOWA) task, a cued-attention task that measures individual differences in spatial attention and orienting proficiency. This analysis identified three profiles that varied substantially in terms of attentional efficiency. The largest of these profiles (“high flexible”, 55%) demonstrated functionally optimal patterns of attentional functioning with relatively rapid, selective, and adaptive orienting responses. The next largest group (“low reactive”, 39.6%) demonstrated low attentional sensitivity with slow, insensitive orienting responses. The smallest group (“high reactive”, 5.4%) demonstrated attentional over-sensitivity, with rapid, unselective and inaccurate orienting responses. A linear mixed effect model and growth curve analysis conducted on 5- to 11-month-old eye tracking data revealed significant stable differences in growth trajectory for each phenotype group. Results from Experiment 2 demonstrated the ability of attentional phenotypes to explain individual differences in general cognitive functioning, revealing significant between-phenotype group differences in performance on a visual short-term memory task. Taken together, results presented here demonstrate that attentional phenotypes are present early in life and predict unique patterns of growth from 5 to 11 months, and may be useful in understanding the origin of individual differences in general visuo-cognitive functioning.

## 1. Introduction

Infant visual attention develops rapidly over the first year of life, significantly altering the way infants respond to visual events, and recent work has demonstrated clear changes in patterns of visual orienting from 5 to 10 months of age [1]. Early in life, visual attention is constrained by biological factors such as immaturity of the retina, optical nerves, and oculomotor system, as well as cortical and subcortical immaturity [2]. However, as the nervous system develops infant attentional capabilities change very rapidly, and this is apparent when observing simple eye movements. This close coupling of eye movements and underlying neural circuitry may make visual orienting tasks an ideal means of assessing functional neural development. For example, even neonates will orient their eyes toward a peripheral stimulus [3]. These eye movements are automatic or reflexive, and are largely mediated by early developing subcortical systems such as the superior colliculus and brain stem [4,5]. Thus, significant deviations in this simple orienting response (e.g., failing to orient, or orienting too readily) may signal underlying neural pathology.

Behaviorally, these reflexive orienting responses involve a quick re-fixation of the eyes to foveate the visual stimulus, and these re-fixations typically occur in a single abrupt eye movement, or saccade. Though young infants demonstrate some degree of hypometria (i.e., consistent undershooting of saccade targets) this effect is most pronounced for large eccentricities and is largely resolved by the time infants are 6–7 months of age [6,7]. As infants develop, the characteristics of saccades continue to change. Over the first months of postnatal life, infants show increasing inhibitory control of reflexive saccades and rapid improvements in volitional saccades. For example, by 3–4 months, infants can learn to suppress reflexive saccades to an attention cue that appears contralaterally to an attractive stimulus [8], and can learn to make anticipatory saccades to expected target locations [9,10,11]. Both of these skills require a sufficiently developed prefrontal cortex, including the frontal eye fields and dorsolateral prefrontal cortex [8,12]. These selective attentional abilities may profoundly influence infant visual learning and memory [13,14,15], and rapid visual orienting is a key component of a flexible and efficient visual cognitive learning system.

Notwithstanding these early constraints on eye movement repertoires, the onset of visual exploration at birth launches a profoundly important period of interactive visuo-cortical development. The importance of these early visual experiences is particularly apparent in cases where visual experiences are atypical. For example, visual deprivation due to congenital cataracts can disrupt processing of high spatial frequencies and object motion [16], face perception [17,18], and cross-modal attention [19], whereas precocious visual experience due to preterm birth alters development of visual attention [20,21], recognition memory [22], and cortical vision [23,24,25] among other things. Clearly, early visual experience is related to the development of multiple visual cognitive skills. However, the interaction between underlying neurophysiology and visual experience may be the true mediator of visual learning. Thus, in order to understand how visual attention contributes to cognitive outcomes, we must find a way to capture the dynamics of this critical brain/behavior interaction before individual differences in cognitive development emerge. If successful, it may be possible to detect sub-optimal attention patterns early in infancy, when intervention efforts are most successful.

Fortunately, links between the characteristics of saccades and underlying neurophysiology are relatively well-understood. Most human research suggests only limited involvement of cortical visual systems like the frontal eye fields and prefrontal cortex prior to six months of age [26]. Saccades prior to that age are likely driven by subcortical structures including the cerebellum, brainstem, and superior colliculus [9,27]. For example, chronic overshooting (hypermetria) of saccade targets is diagnostic of cerebellar dysfunction [28] whereas slow saccades may signify brainstem issues, including the motoneurons and oculomotor nerves [29]. Importantly, although these subcortical eye movement structures develop disproportionately early relative to cortical visual structures [30], the facilitation of orienting achieved by spatial cueing may be insufficient to support higher-order learning and memory [13]. What does this mean for infant eye movement patterns? Limited cortical control of eye movements in the first months of life means most eye movements are predominantly reflexive in response to visual input. However, work with adults suggests that saccades can also be triggered by spontaneous neural activity (i.e., endogenous noise) in the superior colliculus [31,32,33]. These findings are particularly relevant for young infants as neural noise is higher due to limited synaptic development and poor myelination [27,34,35]. In addition, deficits in visual sensitivity the first eight months of life are inversely proportional to levels of intrinsic neural noise [36], though clear individual differences are apparent suggesting endogenous noise may be one key driver of early differences in visual orienting. Although things like neural development, physical maturation, and general health all contribute to this developmental milieu [37,38], visual experience is a critical shaper of continued visual network development [27]. As a result, interactions between neural development and visual experience drive rapid changes in eye movement patterns throughout the first year of life. This is important, as even small physiological differences in eye movement tendencies (e.g., slow saccades or unstable fixations) can qualitatively alter visual learning experiences. These small differences compounded week after week could contribute to the emergence of individual differences in cognitive development more generally. Because physiological differences in eye movement behaviors are relatively easily observed, measures involving simple visual orienting responses may be especially useful. For example, using a simple cued-attention task, it is possible to assess visual performance across multiple domains (speed, accuracy, spatial attention, saccade inhibition, etc.). By taking a multivariate approach, it may be possible to characterize each infant’s unique attentional style or *phenotype*, and determine if phenotypic differences are related to individual differences in cognitive performance. To accomplish this, we will use the Infant Orienting with Attention (IOWA) task, a cued-attention task that measures individual differences in spatial attention and orienting proficiency [1,20].

**Figure 1:**
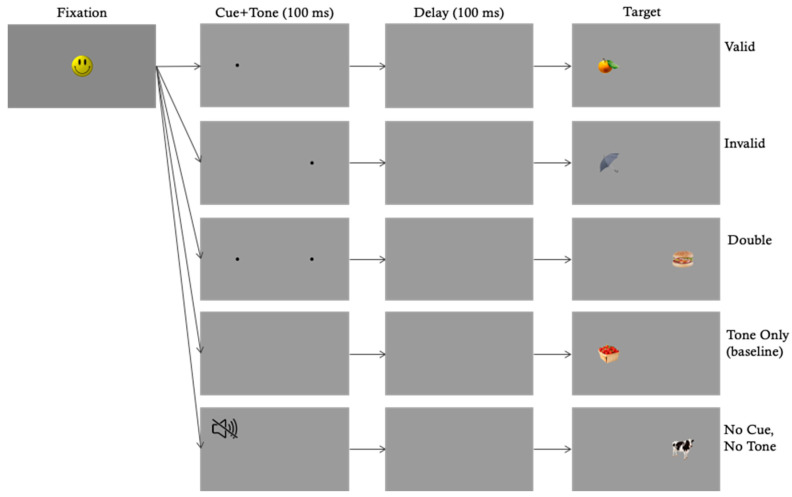
Experimental and control conditions from the Infant Orienting with Attention (IOWA) Task [1].

The IOWA task is based on well-documented spatial cueing effects [8,9,10,11,39,40,41], and provides a set of continuous measures based on both overt shifts of attention (i.e., eye movements), and covert shifts of attention (i.e., spatial cueing effects) [42]. Primary dependent measures consist of saccadic reaction time to a peripheral target (SRT), and orienting accuracy across a variety of cue conditions, including congruent (valid spatial precues), incongruent (invalid spatial precues cues) and neutral cued attention trials (double spatial precues), along with baseline control trials. Accuracy and SRT can be used to create a series of difference scores to assess the development of spatial attention, orienting speed, saccade inhibition and visual competition. These scores can further be normalized to facilitate the comparison of spatial attention both within an age (i.e., individual differences), and between ages (i.e., developmental differences) despite differences in baseline orienting speed (see Figure 1).

The task has been used to assess attentional functioning in both full-term [1] and preterm infants [20], as well as infants in rural Malawi [43] making it an ideal candidate for examining both overall development, and the emergence of individual differences. In general, results from this task highlight substantial improvements in orienting speed, coupled with increasing facilitation from congruent spatial cues, decreasing interference from incongruent spatial cues, and increases in visual competition [44] from 5- to 10-months-of-age in full-term infants [1]. Preterm infant findings reveal substantially less facilitation and substantially more interference than their full-term counterparts, which suggests relatively poor attentional selectivity, saccade inhibition, or both [20]. Though previous results show developmental improvement in overall orienting speed, accuracy, and spatial attention, not all infants showed this pattern of responding. Some infants showed fast orienting speeds and strong spatial cueing effects but made numerous errors, suggesting poor saccade inhibition for the invalid cues. Still others demonstrated relatively weak spatial cueing effects and slow orienting speeds but made few errors, suggesting relatively underdeveloped spatial attention mechanisms and slow processing speed. Thus, assessing stability using cued-attention tasks requires a multivariate approach, and this may be why there is relatively little longitudinal research examining spatial cueing effects in infants. If individual differences are stable over time, they could radically alter both the quality of visual input detected and subsequently fixated, and the quantity of visual events that elicit visual exploration.

Previous research demonstrates that visual behaviors (e.g., fixation rate, look duration, peak look, shift rate, etc.) and attentional biases are relatively stable during the first year of life [22,43,45,46,47], and show moderate cross task stability within both cognitive and social contexts [48]. Though several of these tasks relied on holistic measures of attention such as peak look duration and switch rate in the context of longer-term memory tasks, the important commonality across all of these measures is underlying patterns in general orienting behavior. Based on these findings, we posit that individual differences in attentional phenotypes will appear early in development and remain relatively stable over time. Moreover, to the extent that individual differences in attentional phenotype mediate the relationship between visual experience and subsequent visual cognitive development, we predict that attentional phenotypes will be related to performance differences on a qualitatively different cognitive task. In Experiment 1, we tested full-term infants longitudinally at 5-, 8-, and 11-months of age, and conducted a latent profile analysis (LPA) on 11-month-old IOWA scores to identify distinct attentional subtypes. We then confirmed the stability of these profiles by conducting growth trajectory analyses from 5 to 11 months of age. In Experiment 2, we examined the relation of each profile identified in Experiment 1 (i.e., attentional phenotype) to cognitive development more generally, by examining performance on a separate visual short-term memory (STM).

## 2. Experiment 1

The goal of Experiment 1 was to determine if attention, speed, and accuracy measures taken from the IOWA task could be used to identify attentional phenotypes, and to determine if these attentional phenotypes predicted distinct growth trajectories from 5 to 11 months. To accomplish this, we recruited infants at 5 months of age, and tested them in the IOWA task at 5, 8, and 11 months of age. Next, we conducted a latent profile analysis (LPA) on six key IOWA attention scores to classify 11-month-old infants into distinct profiles. We chose to conduct these analyses at 11 months for several reasons: Eye-tracking data from older infants tends to have a higher signal to noise ratio than younger infants [49], older infants were more likely to stay engaged in the task, and previous research suggested attention score variability was highest in older infants [1]. Lastly, linear mixed effects models (LME) with conditional growth curve analyses were used to assess the stability phenotype group cohesion from 5 to 11 months of age.

### 2.1. Methods

#### 2.1.1. Participants

All infant names were obtained from the Tennessee Department of Health and were born full term with no reported birth defects or vision problems. The current sample was drawn from an ongoing longitudinal study of infant cognitive development. Infants were recruited at 4 months of age and invited for lab visits at 5, 8, and 11 months of age. The original full sample included 117 5-month-olds (*M_days_* = 155.44, *SD_days_* = 8.71), 120 8-month-olds (*M_days_* = 245.78, *SD_days_* = 8.95), and 118 11-month-olds (*M_days_* = 335.9, *SD_days_* = 8.89). Twenty infants were excluded from this original sample due to fussiness or fatigue (*N* = 10 at 5 mo, *N* = 1 at 8 mo, and *N* = 4 at 11 mo), experimenter error or equipment failure (*N* = 2 at 8 mo, and *N* = 3 at 11 mo). All methods and procedures were approved by East Tennessee State University IRB (protocol number: 0314.29s, date: 2/12/2014).

From this complete sample, we selected all infants who successfully completed the IOWA task at their 11-month visit and at least one other visit. This resulted in a final sample of 111 11-month-olds months (*M*_days_ = 335.82, *SD*_days_ = 8.58, 62 males and 49 females), 79 of whom participated at 8 months (*M*_days_ = 246, *SD*_days_ = 7.99, 49 males and 30 females), and 69 of whom participated at 5 months (*M*_days_ = 157.81 days, *SD*_days_ = 9.37, 38 males and 31 females). Of the 111 selected infants 97 were White, 1 was Black, 9 were multi-racial, 1 was Middle Eastern, and 3 parents chose not to respond. In addition, 102 infants were Non-Hispanic, 4 were Hispanic, and 5 parents chose not to respond. Overall, 99.10% of the mothers had graduated high school, and 58.56% had at least a bachelor’s degree. For every visit, parents received a $20 gift card and infants received a small toy. Transportation and sibling childcare for the visits were provided if needed.

#### 2.1.2. Stimuli and Procedure

A Tobii TX-300 eye tracker (Tobii Technology, Danderyd, Sweden) was used to present the stimuli on a 23” monitor with a viewable surface of 45.5° (w) by 26.76° (h) at a distance of 65 cm. A Cedrus StimTracker was used to precisely mark stimulus events via pixel photodetection, and all latency calculations were conducted with respect to these event marks. Infants were tested in a dimly lit room on their parent’s lap, and continuous gaze was recorded as they viewed each trial. Prior to data collection, each infant was calibrated using a 5-point calibration scheme, and testing did not proceed until reasonable accuracy was obtained for all 5 points. (Note that the Tobii Studio calibration routine relies on qualitative validation after calibration. After calibration, fixation stimuli were re-presented to each of the 5 calibration points, and calibration precision and accuracy were indicated graphically as deviance from stimulus center. Any points that deviated substantially beyond the 1° boundary were subsequently resampled and revalidated. This procedure continued until all five points showed good precision and accuracy). An observer seated out of sight would initiate a trial when the infant was judged to be fixating the central attention-getter (i.e., a musical dancing smiley face, see Figure 1). Each trial consisted of a 100 ms precue consisting of a spatial cue (1° black dot) presented to the left or right of central fixation. For some conditions, this spatial cue was accompanied by a 100 ms auditory tone (500 Hz sine wave). The precue interval was followed by a 100 ms blank interval, followed immediately by the presentation of a target either in the same location as the spatial precue (i.e., valid cue) or contralateral to the spatial precue (i.e., invalid cue). Targets consisted of 5° (w) by 5° (h) digital images of colorful everyday objects and were sampled without replacement from a pool of 182 images. Targets and spatial cues were each presented 11° degrees to the left or the right of fixation. Experimental conditions were created by manipulating the validity of the cues as follows: The valid cue condition contained a spatial + auditory cue presented in the same location as the target. The invalid cue condition contained a spatial + auditory cue presented contralaterally to the target, and the double cue condition contained spatial + auditory cue presented simultaneously to the left and right of fixation followed by the target. Two additional control conditions were created as baseline measures of orienting speed and included the tone cue condition which contained an auditory cue but no spatial cue, and the no cue condition which contained neither an auditory nor a spatial cue. Targets remained visible for 1000 ms after which the attention-getter reappeared, and the next trial began. Each block contained two of each cue condition (one with target on left, and one with target on right) presented in random order, and infants saw up to 12 blocks (120 trials). Testing continued until infants lost interest or became fussy, and all infants completed at least 30 trials (*M_trials_* = 78.07, *SD_trials_* = 22.28).

#### 2.1.3. Measures

A 15° (w) by 15° (h) interest area was drawn around both left and right target locations, and the first fixation within 1000 ms of target appearance was coded as the response. These interest areas were chosen to closely mirror human standards of movement detection, and as such were sufficiently large to detect looks that fall outside of the target stimulus due to calibration error. As in Ross-Sheehy et al. 2015, only trials in which the infant was fixating center at target onset were included, and saccades that occurred within 100 ms of target presentation were discarded as anticipatory. Primary dependent measures included saccadic reaction time to the target (SRT), and accuracy (correct = first saccade to the target). Only correct looks were used in reaction time measures. Infants who could not be calibrated or whose gaze was lost for more than 50% of their trials (*N =* 42 of 239 sessions) were frame-by-frame coded offline from video captured at 60 fps (i.e., 16.6 ms/frame). To ensure human observers and eye tracking measures were qualitatively and quantitively comparable, we frame-by-frame coded an additional 25% of the 11-month-old infants with high quality eye tracking and compared human-derived measures with eye tracking-derived measures. Percent agreement for direction of first look was high (95%), and mean SRT differences were reasonably low (*M* = 48.68 ms, *SD* = 70.43). This is important and demonstrates frame-by-frame coding may be a reasonable alternative when eye tracking is infeasible or calibration accuracy is questionable.

A series of three different scores were created (correct trials only) to assess the magnitude of spatial cueing effects. These attention scores were then normalized using each infant’s baseline orienting speed (mean SRT for tone cue condition) to ensure that spatial cueing effects were not confounded with general differences in orienting speed. These scores included Cue facilitation ((baseline SRT-valid SRT)/baseline SRT), with higher scores indicating faster SRTs for valid cues relative to baseline; Cue interference ((invalid SRT-baseline SRT)/baseline SRT), with higher scores indicating slower SRTs for invalid cues relative to baseline; and Cue competition ((double SRT-valid SRT)/baseline SRT), with higher scores indicating slower SRTs for double cues relative to valid cues. Three additional scores were calculated to capture overall differences in orienting speed and accuracy. These are mean saccadic reaction time (mean SRT across all conditions for correct trials only), with higher scores indicating slower overall orienting speed; Task error (1- average (double % correct and invalid% correct)), with higher scores representing more error in conditions that contained visual competition (invalid and double cue conditions). Note that these errors are task relevant, and can occur if infants are fast enough processors that they respond to the cue prior to the appearance of the target. The last score is Baseline error (1- average (valid % correct, tone % correct, no cue % correct)), with higher scores representing more errors in conditions with no visual competition (valid, no cue, tone cue). Note that performance is typically near ceiling for these conditions, thus a high baseline error score can be a marker of off-task behavior, calibration noise, or visual deficits.

Although the goal of the current work is to examine individual differences, an outlier analysis was conducted to ensure that the six attention scores did not contain implausible scores, or values that were inconsistent with observed trends in the data. Aberrant scores due to eye tracker or calibration noise should produce extreme values across all attention scores, therefore infants with plausible scores and fewer than 4 outlier values should be considered valid. Of the 11 subjects with outlier scores (i.e., scores >3rd quartile + 1.5 times the interquartile range, or <1st quartile − 1.5 times interquartile range), 9 infants contributed only a single outlier data point, 1 infant contributed 2 outlier data points, and 1 infant contributed 3 outlier data points. Thus, all infants were included in subsequent analyses.

#### 2.1.4. Data Reduction

Binocular gaze was sampled continuously at 300 Hz throughout the testing session, and saccades and fixations were parsed using a velocity threshold filter (Tobii I-VT fixation filter, Tobii Technology, Danderyd, Sweden) [50] with the following parameter settings: Left and right gaze was averaged, interpolated across gaps of 75 ms or less, and smoothed using 3-sample moving average. A velocity calculator window of 20 ms was used, and the threshold for calculating a saccade was 30 degrees/second. Short fixations were merged (<75 ms, <5°) and looks shorter than 60 ms were discarded.

### 2.2. Results

**Figure 2:**
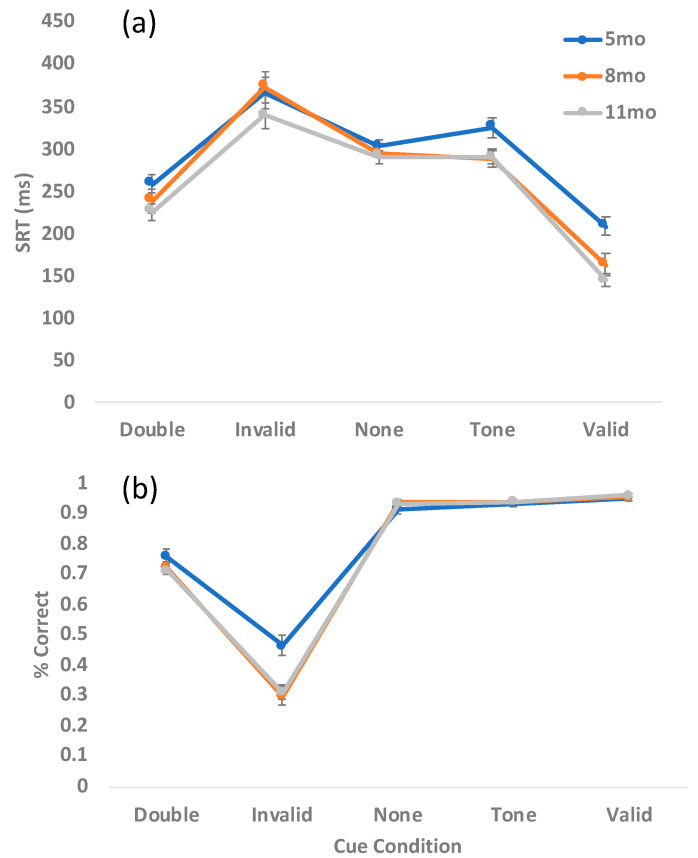
Raw saccadic reaction time (SRT) data (**a**) and percent correct (**b**) by age. Error bars represent +/− SEM.

Raw SRT and percent correct data are presented in Figure 2. In general, infants were fastest and most accurate for the valid cues, slowest and least accurate for the invalid cues, with particularly strong cueing effects at 8 months. Developmentally, SRT appears to decrease with age, with particular improvements from 5 to 8 months. Importantly, the rate of SRT improvement appears to outstrip the development of saccade inhibition as evidenced by increasing error rates for the older infants-particularly for the invalid and double cue conditions. This pattern of development replicates previously published findings [1].

Next we calculated six composite attention scores using raw SRT and percent correct data (see Measures above). Facilitation and interference scores measure the strength of spatial cueing effects for the valid (former) and invalid (latter) cues. Any score larger than zero demonstrates the presence of spatial cueing effects, and higher scores represent stronger effects. The competition score is a measure of the visual competition caused by the presentation of two simultaneous cues in the double cue condition. The stronger the competition effect, the larger the score. As its name suggests, mean SRT is simply an average of each infant’s SRT across all conditions providing a robust assessment of general orienting speed. Baseline error reflects random trial to trial error, and task error reflects errors produced in conditions that require saccade inhibition. High baseline errors may suggest an infant is off task or has visual deficits, whereas high task error typically indicates that the infant is engaged and is very sensitive to spatial cueing effects. Although this may seem counterintuitive, errors occur when an infant is fast enough that they have begun saccade execution prior to the onset of the target. However it also suggests that although spatial attention and SRT systems are well-developed, the infant may yet lack the cortical attentional control necessary to inhibit the reflexive saccade to the invalid cue. It stands to reason that as prefrontal cortex continues to develop, task error should drop.

The primary aims of Experiment 1 are to: (1) Determine if attentional phenotypes can be identified using the six IOWA attention scores (see Measures above), and (2) assess the ability of these attentional phenotypes to predict group differences in developmental growth trajectory. We will address aim 1 by conducting a latent profile analysis (LPA) on IOWA attention scores, and will address aim 2 by conducting linear mixed effects (LME) models on the 11 month cluster data to predict growth trajectories by cluster from 5 to 11 months of age.

#### 2.2.1. Assessing Attentional Phenotypes at 11-Months-of-Age

The six attention scores described above capture individual differences in spatial cueing, speed of orienting, and saccade inhibition. While previous work has used these scores to probe development cross-sectionally, here we are using them longitudinally to assess stability of patterns of attentional performance over time. In this way, it may be possible to identify which scores are most likely to contribute to stable attentional phenotypes. As a first step, we submitted the 11-month-old IOWA attention scores to an exploratory LPA using Mplus (version 8.4, Muthén & Muthén, Los Angeles, LA, SA) [51] and the MPlusAutomation package (version 0.7-3) for R [52]. (Note that 7 of the 111 subjects made no errors and thus had no interference score. These scores were addressed in MPlus using maximum likelihood estimation [53]). Latent profile analyses are essentially latent cluster analyses (LCA) with continuous outcome measures [54]. As is the case with LCA, LPA can be very useful for identifying behavioral patterns or clusters across multiple dependent measures, making it an ideal tool identifying attentional phenotype groups.

**Figure 3:**
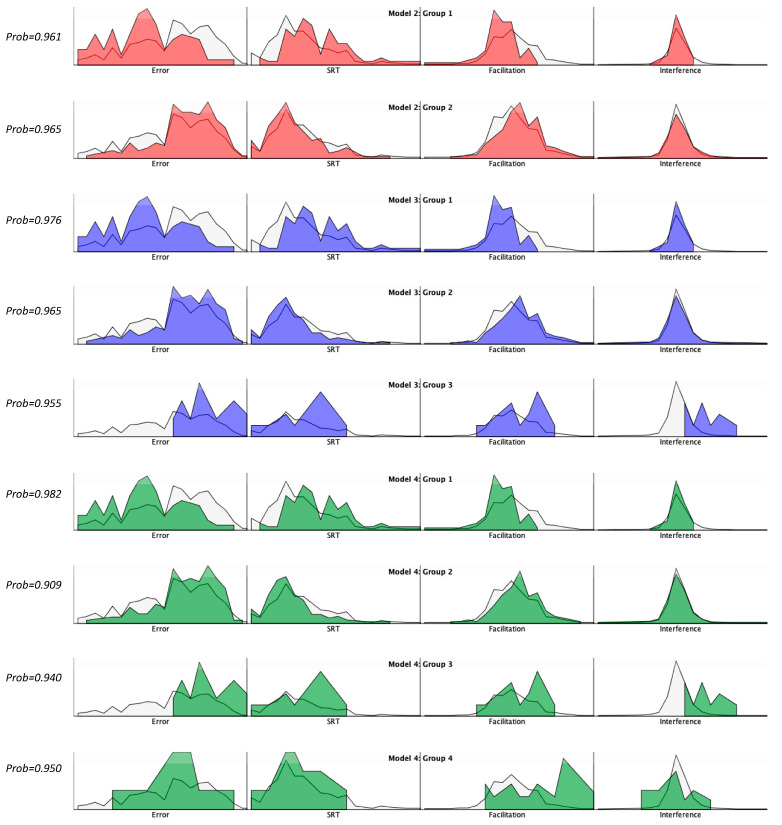
Frequency histograms for Models 2–4 as a function of latent profile group. Percent counts in color overaII means in light grey. The four IOWA task performance variables include task error (Error), saccadic reaction time (SRT), cue facilitation (Facilitation), and cue interference (Interference). Posterior probabilities for classification (Prob) indicated to the left or each group.

As is typical for exploratory LPA analyses, models were computed for profile sizes 1–5, and the 2, 3, 4, and 5 profile models all produced good model fits. Although theory and interpretability should always guide model choice, this is especially important when multiple models provide good statistical fits [54,55] as there is currently no single accepted approach to model selection [56,57]. Lo-Mendell Ruben (LMR) likelihood ratio tests were conducted to examine goodness of fit for each model (k) compared to the previous simpler model (k-1). Results revealed significantly better model fits for 2-profile model relative to the 1-profile model. Results for the 3-, 4-, and 5-profile models did not differ significantly suggesting the minimum complexity required for adequate fit to be 2 profiles, and that the 2-, 3-, 4-, and 5-profile models all produced good fits. We next examined log likelihood (logLik), Akaike information criterion (AIC), Bayesian information criterion (BIC) and entropy to determine which models resulted in overall best fits (Table 1). Log likelihood is a measure of goodness of fit, with higher numbers representing better model fits. Both AIC and BIC incorporate a penalty on model complexity to help prevent “overfitting”, the arbitrary addition of profiles to improve model fits without regard to interpretability or theoretical support. Low values for both AIC and BIC indicate best model fits. Note that although both AIC and BIC are commonly used for model selection, BIC tends to be more accurate for models with continuous variables [57]. Entropy is a widely used criterion of classification accuracy, with higher values indicating more precise assignment of individuals to profiles. Both BIC and entropy strongly favored the 4-class model, whereas the AIC favored the 5-class model (Table 1), thus the more complex 5-profile model was eliminated from further consideration. This left as candidates the 2-, 3- and 4-profile models, each of which produced excellent statistical fits, though both the 3-profile and 4-profile models produced substantially better fits than the 2-class model. Three key aspects were examined when making our final model selection: (1) Which model produced readily discernible and interpretable profiles, (2) which model produced profiles that preserved the most between-group variability, and (3) which model provided the most parsimonious solution. Figure 3 presents frequency histograms for each of these three candidate models, along with posterior probabilities for classification. Though the 2-profile model is the simplest, the 3-profile model had stronger fit statistics, and produced three readily distinguishable profiles. The 4-profile model has best overall model fits, but group 4 was not qualitatively different from group 2 making interpretation problematic. Finally, posterior probabilities for classification certainty were highest for model 3. Interestingly, the group 3 composition was exactly the same for both the 3- and 4-profile models. This along with its relatively high posterior probability of classification bolstered our confidence that this group captured a small but meaningful behavioral trend. Thus model 3 appeared to provide the best balance between model fits, profile distinguishability, and parsimony (see Table 2 for means and SD by profile).

Group 1 (*N* = 44) is characterized by weak spatial cueing effects, slow reaction times, and very low error rates. This cluster is labeled low reactive, as these infants tend to be relatively insensitive to the effects of the cue. In addition, low error rates likely reflect slow processing speed so it is unclear if these infants are capable of saccade inhibition. Group 2 (*N* = 61) is characterized by strong spatial cuing effects, rapid reaction times, and moderate to low error rates. This cluster is labeled high flexible, because these infants show strong cueing effects when the cue is helpful, and sufficient saccade inhibition to prevent errors when the cue is distracting. These infants are also reasonably fast processers and “orienters”. Thus, the high flexible phenotype demonstrates an ideal pattern of responding. Group 3 (*N* = 6) is characterized by strong spatial cueing effects, fast reaction times, and high error rates. This cluster is labeled high reactive, as they seem to demonstrate hypersensitivity to the cue. The effects of this oversensitivity are further exacerbated by their fast orienting speeds, and low saccade inhibition. This may be the least developmentally advantageous phenotype, as oversensitivity to peripheral events suggests they may struggle with visual learning tasks that require sustained fixation or saccade inhibition [13,14]. Despite the small number of participants in the high reactive group, at >5% of the sample they were sufficient in number to warrant inclusion, and their presence improved overall model fit, entropy, and posterior probabilities for the remaining two profiles. In addition, as can be seen in Figure 3, the pattern of orienting is highly consistent across individuals within this group, and is consistent with previously predicted effects of fast processing and strong spatial attention without the benefit of saccade inhibition [1].

Two additional factors were examined to ensure all three phenotypes reflected genuine behavioral differences rather than low-level confounds: Task engagement as indexed using trial counts, and calibration accuracy as indexed by the number of infants in each group requiring hand coding. Neither trial count (*Mean_trialHF_* = 80.08, *SD_trialHF_* = 21.65; *Mean_trialLR_* = 83.07, *SD_trialLR_* = 24.07; *Mean_trialHR_* = 79.17, *SD_trialHR_* = 21.85), nor calibration accuracy (*Coded_HF_* = 26, *Coded_HR_* = 2, *Coded_LR_* = 14) differed substantially from overall phenotype proportions. In addition, these general cluster proportions were maintained across all three ages: High flexible: *N* = 38, 45, and 61 for 5-, 8-, and 11-month-olds, respectively. High reactive: *N* = 4, 3, and 6 for 5-, 8-, and 11-month-olds, respectively. Low reactive: *N* = 17, 21 and 44 for 5-, 8-, and 11-month-olds, respectively.

#### 2.2.2. Assessing the Stability of Attentional Phenotypes

**Figure 4:**
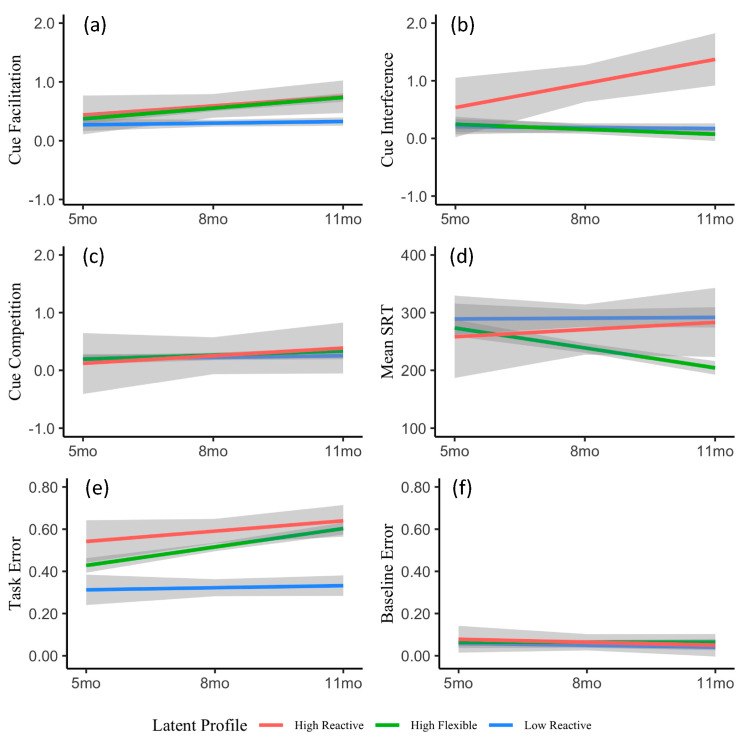
Fitted growth trajectories by age, attention score, and latent profile. Linear mixed-effects (LME) models based on age and phenotype (model 2) accounted for significantly more variance than age-only models (model 1) for four attention scores: Cue facilitation (**a**), cue interference (**b**), mean SRT (**d**), and task error (**e**) all *p* < 0.001. In contrast, model 2 did not differ from model 1 for either cue competition (**c**) or baseline error (f). Grey shading indicates 95% confidence interval.

To further explore the stability of these phenotypes over the first year of life LME models were used to fit conditional growth curves by profile from 5 to 11 months (R package: nlme). Separate LME models were conducted for each of the six attention scores, with age (5, 8, or 11 months) and profile (high reactive, low reactive, or high flexible) as fixed factors, and subject as a random factor. This allows us to assess the ability of profile membership to explain growth trajectory differences from 5 to 11 months (see Table 3). In general, results revealed three attention scores with significant profile effects. These are interference, task error, and mean SRT. Although these results demonstrate clear profile effects for at least three attention scores, our next analysis sought to more directly assess the ability of profile to explain differences in growth trajectory over-and-above development alone. To accomplish this, we conducted separate ANOVAs for each attention score comparing our previous age by profile LME (i.e., model 2) to a time-only baseline model (i.e., model 1). Results revealed significantly better model 2 fits for cue facilitation, χ^2^(4) = 55.01, *p* < 0.001; cue interference, χ^2^(4) = 46.07, *p* < 0.001; task error, χ^2^(4) = 93.06, *p* < 0.001; and mean SRT, χ^2^(4) = 65.43, *p* < 0.001 (see Table 4 for model tests and Figure 4 for fitted growth trajectories). These findings suggest that developmental trajectories vary significantly by attention phenotype for four IOWA attention scores: Cue facilitation, cue interference, task error, and mean SRT. In addition to helping identify the four IOWA scores that were most useful in differentiating early predictors of later individual differences in visual orienting, this analysis provides very strong validation of the 3-profile model. Specifically, although the high reactive phenotype group is small, group cohesion was strong, and groupwise trends were clear at all three ages for both cue interference and task error. Moreover, the finding that high reactive infants differed only on scores that assess visual competition further supports the hypothesis that phenotypic differences were due in part to relatively low saccade inhibition. Clear differences in growth trajectory were also apparent for both the high flexible and low reactive infants, particularly for the cue facilitation, mean SRT, and task error. If these phenotypes merely reflected random performance factors, general maturation, or eyetracker noise, we did not expect phenotype group (model 2) to do a better job of predicting growth than the time only baseline model (model 1).

### 2.3. Discussion

The aims of Experiment 1 were twofold: First to determine if attentional phenotypes could be identified using the six IOWA attention scores, and second to assess the ability of these attentional phenotypes to predict group differences in developmental growth trajectory. In addition to replicating previous findings [1], results from both our LPA and LME analyses suggest that distinct attentional phenotypes are present and readily identifiable at 11 months of age, and that these phenotypes predict distinct growth trajectories for four of the six IOWA attention scores tested: facilitation, interference, mean SRT, and task error. Model fits presented in Figure 4 reveal distinct growth trajectories for each attentional phenotype, and the patterns of difference may help elucidate mechanisms that underlie attentional proficiency. For example, although both the high flexible and high reactive infants show increasing sensitivity to valid spatial cues (cue facilitation), only the high reactive infants become increasingly able to resist the distraction of an invalid cue (cue interference). This suggests that high reactive infants may lack development in frontal eye fields and other cortical sources of saccade inhibition. Consistent with that interpretation, high reactive infants consistently demonstrate very high task error rates, and this effect does not appear to change from 5 to 11 months. This high reactive phenotype, though small in number, represents an important developmental profile, as it may signify a relatively unselective attentional orienting system. Future work will determine if these infants are at greater-than-average risk for attentional dysfunction or distractibility later in life. Although it may seem counterintuitive that high flexible infants demonstrate increasing task errors, this appears to be related to concurrent decreases in reaction time (mean SRT). It is likely the case that the mechanisms that subserve orienting speed (e.g., spatial attention, processing speed, saccade programming, saccade execution, etc.) mature more rapidly than saccade inhibition mechanisms, resulting in what appears to be a speed/accuracy tradeoff. Interestingly, the low reactive infants show very little developmental change from 5 to 11 months. This phenotype demonstrates low spatial attention (facilitation, interference) and slow mean RTs. Though their error rates are relatively low, this is most likely due to slow saccade programming/execution and poor spatial attention rather than high inhibitory control of saccades. Thus, low reactive phenotype may be somewhat delayed relative to the high flexible phenotype.

## 3. Experiment 2

Experiment 1 demonstrated that attentional phenotypes are present early in life and produce qualitatively and quantitatively discernible patterns of visual orienting. However, it is currently unclear if these phenotypic differences are meaningful outside the context of a rapid visual orienting task. Further, it is currently unclear if the behavioral tendencies evidenced by our three phenotype groups will manifest in other more cognitive tasks. The goals of Experiment 2 are to: (1) Determine if attentional phenotypes produce detectable differences in eye movement behaviors on a qualitatively different kind of task, and (2) to determine if attentional phenotypes produce detectably different patterns of performance on a cognitive task. The cognitive task we have chosen is a visual STM task [58]. There are several reasons for this selection: First, previous research suggests successful change detection, a measure of visual STM fidelity, may be influenced by attention in infants [15] and adults [59,60], even in the absence of overt orienting responses. Second, whereas our attention task was designed to elicit rapid and reflexive orienting responses, our visual STM task was not. The simultaneous appearance of multiple stimuli typically inhibits rapid reflexive orienting [39,40,61,62], and the slower task intervals are sufficiently long to allow for multiple saccades and fixations. Third, STM is a key cognitive ability and a strong predictor of overall academic achievement [63,64,65]. Finally, using a task that taps fundamentally different visual-cognitive skills provides a strong test of our hypothesis that persistent individual differences in eye movement tendencies underpin performance on all visual tasks. Though infant change detection tasks incorporating a dual-screen procedure typically use “change preference” to infer change detection [66] (i.e., longer look durations to the changing array versus the unchanged array), previous work using single-screen “one-shot” procedures suggests change preference (i.e., longer fixations to the changed feature versus the unchanged features) may be confounded with fixation behavior during encoding for both infants and adults [58]. That is, infants and adults who simply made more eye movements were more likely to have fixated the to-be-changed item during encoding resulting in higher change preference scores. Thus in this one-shot context, change preference may be more reflective of scanning behavior than STM capacity, per se. Importantly, lower-level visual dynamics such as visual scanning and total looking did appear to vary with STM fidelity for both infants and adults. Thus, we chose these measures as a strong test of our hypothesis that attentional phenotypes underpin individual differences in cognitive functioning above-and-beyond what can reasonably be attributed to simple visual orienting differences.

### 3.1. Methods

#### 3.1.1. Participants

The final sample consisted of 102 infants tested in Experiment 1 at 11 months (A full analysis of these visual STM results have been reported elsewhere [58]. Here we are simply using visual STM performance to test the hypothesis that attentional phenotype influences cognitive performance more generally). An additional 9 infants were excluded from this analysis due to fussiness or general fatigue (*N* = 4), or experimenter error (*N* = 5). The resulting attention phenotype counts were as follows: high reactive (*N* = 5), low reactive (*N* = 42), and high flexible (*N* = 55). Although the sample for the high reactive phenotype is small, analyses presented in Experiment 1 demonstrate that excluding these infants resulted in significantly reduced model fits. Thus, although they are few, they do appear to represent an important phenotype profile.

#### 3.1.2. Stimuli and Procedure

**Figure 5:**
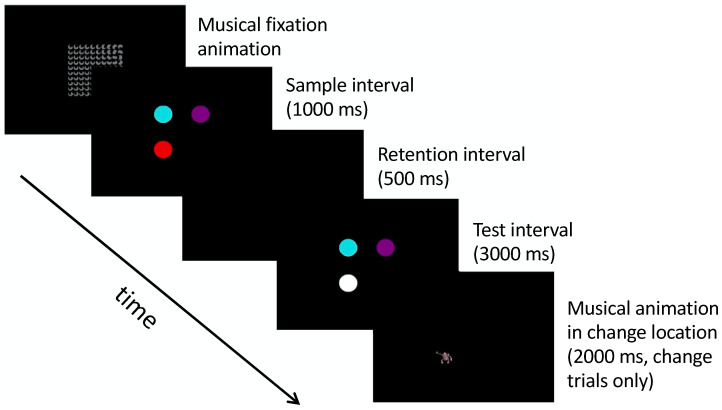
Example of a set size 3-change trial. No change trials were identical to change trials with two exceptions: First, the test array matched the sample array, and second, the test array was followed by a 2000 ms blank interval rather than a musical reward animation.

General eye tracking procedures were identical to Experiment 1, and details of the visual STM task will be briefly summarized below [58]. Stimuli consisted of arrays of colored circles, presented against a black background 8° from central fixation at 45°, 135°, 225°, and 315°. All circles measured 5° in diameter, and total eccentricity measured 15.5° (Figure 5). Set size (1–4 circles) and condition (change, no-change) were tested within subjects, resulting in 8 unique trial types. These trials were presented randomly within each block for up to 12 blocks (96 trials max) or until infants lost interest or became fussy. Average trial counts by attention phenotype were as follows: High flexible (*M_trials_* = 29.23, *SD_trials_* = 10,074), high reactive (*M_trials_* = 27.75, *SD_trials_* = 7365), and low reactive (*M_trials_* = 33.33, *SD_trials_* = 14,272).

Each trial began with a dynamic central fixation stimulus that both attracted attention and helped increase between-trial distinctiveness. Once the infant was judged to be fixating, the trial was started, and infants were presented a 1000 ms sample array comprised of 1–4 colored circles, followed by a 500 ms blank retention interval, and finally a 3000 ms test array that was either identical to the sample array (no-change trials), or included a color change in one random location (change trials). All change trials were followed by a 3 s audiovisual animation in the location of the change. This reinforcing stimulus served to increase the likelihood of fixating the changed circle, and generally keep interest high. Primary dependent measures included total look duration (i.e., the summation of all fixations during the test interval), and switch count (i.e., the number of times an infant looked from one circle to another during the test interval).

#### 3.1.3. Data Reduction

Data reduction procedures were identical to Experiment 1.

### 3.2. Results

Previous research using this task [58] suggested that switch rate and duration of looking during the test array were particularly good indicators of adult performance. Specifically, adults made fewer switches and produced shorter overall look durations during the test array for correct trials (i.e., selected “same” for non-changing trials, and “different” for trials in which a single color changed). Although we cannot assess accuracy in infants, previous results indicate that low-level visual behaviors such as switching and look duration are influenced by higher cognitive processes such as change detection. Thus, it should be possible to use these general behaviors to assess relative performance for each of our three phenotypes. If attention phenotypes reflect fundamental and persistent differences in eye movement tendencies, then performance on this visual STM task should vary by phenotype. However, given the particularly small sample size for the high reactive phenotype, we first conducted omnibus nonparametric tests to determine if group level differences were present for each measure. To accomplish this, we conducted an independent samples Kruskal–Wallis Test on individual look duration and switch counts averaged across trial, such that each participant contributed 1 sample for each condition and set size. This maximized power to detect group differences while minimizing between-subject variability due to individual differences in trial count. Results revealed significant group effects for switch count, χ^2^(2) = 14,312, *p* = 0.001, with mean score ranks of 434.49 (high flexible), 434.98 (high reactive), and 371.32 (low reactive), and for total looking, χ^2^(2) = 51,897, *p* < 0.001, with mean ranks of 458.28 (high flexible), 457.65 (high reactive), and 337.46 (low reactive). These results demonstrate significant between-group differences for both measures, thus subsequent analyses were carried out on both switch count and total looking.

**Figure 6:**
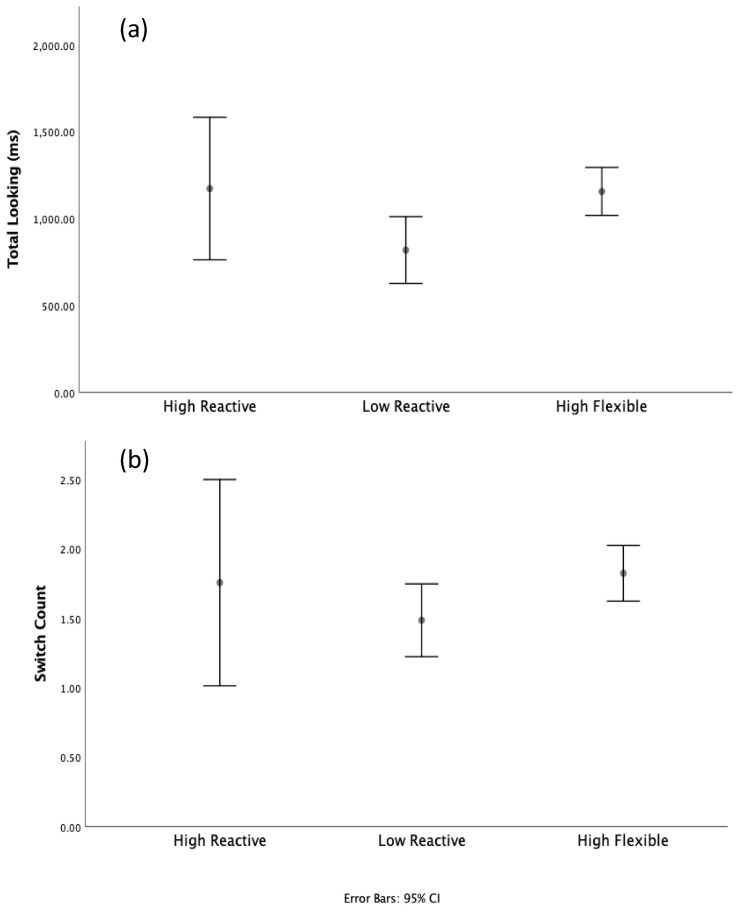
Mean look duration (**a**) and switch count (**b**) as a function of attention phenotype. CI: confidence interval.

To explore the specific group differences with look duration and switching, independent sample *t*-tests were conducted. Although this analysis is somewhat robust to unequal samples and small sample sizes, we opted to incorporate a bootstrapping procedure. This approach allowed us to derive unbiased sample statistics based on an estimated sampling distribution created through the random sampling of group means (sample count = 5000, confidence interval = 95%). This allows for robust hypothesis testing when normality of distributions cannot be assumed. An added benefit of this approach is that it is robust in the face of small sample sizes and is relatively insensitive to outliers. As with our nonparametric testing, we averaged participant data across the trial to reduce between-subject variability, and additionally averaged data across condition and set size to reduce within-subject error due to individual differences in visual STM capacity. Results for total look durations revealed that the low reactive infants looked significantly less than both the high flexible infants, *t*(95) = −2944, *p* = 0.004 and the high reactive infants, *t*(45) = 1253, *p* = 0.038. No other comparisons for look duration were significant (Figure 6). Results for switch count revealed that high flexible infants made significantly more switches than low reactive infants, *t*(95) = −2099, *p* = 0.038. No other comparisons for switch count were significant (Figure 6).

The previous analyses demonstrated significant between-group differences for measures of look duration and switch count. The goal of the next series of analyses is to assess visual STM performance for each group by comparing switch rate and total looking across set size and condition. Note that in this task evidence for change detection (a marker of visual STM accuracy) should manifest as longer looking to the change trials than to the no change trials for set sizes that are within STM capacity limits [66,67]. To examine this, we conducted separate 2 × 4 repeated measures ANOVAs for look duration and switch count with condition (change, no change) and set size (1, 2, 3, or 4) as within subject variables. Although we could have incorporated attentional phenotype as a between-subjects factor, our previous analyses already demonstrated clear between-group effects. Thus, to protect familywise error, we opted to conduct separate ANOVAs for each phenotype. Due to the small number of high reactive infants, we conducted an effect size analysis and determined that 0.477 is the minimum effect size necessary to reject the null (*N* = 5, alpha = 0.5, power = 0.8). Because this is a large effect for an *F*-test, results for the high reactive phenotype should be interpreted with extreme caution.

**Figure 7:**
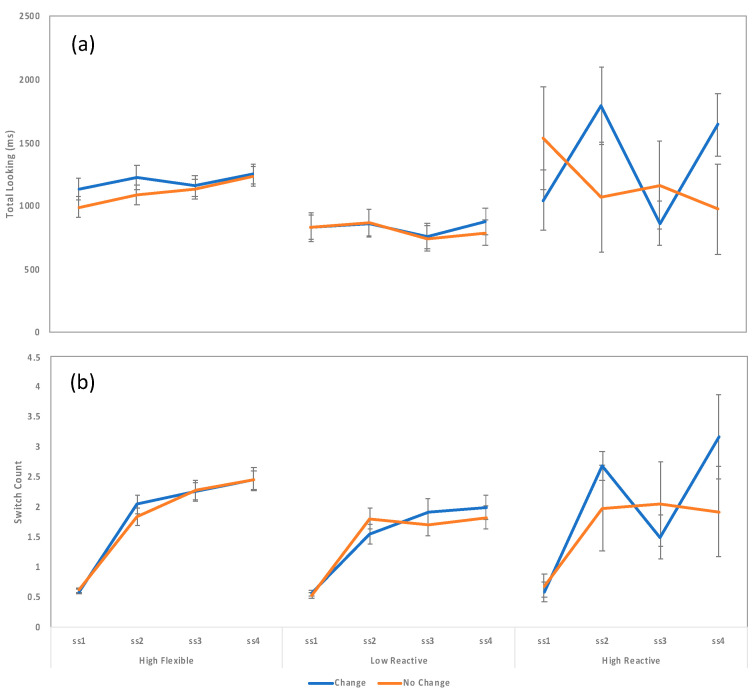
Mean total looking (**a**) and switch count (**b**) during the test array as a function of attention phenotype, change status and set size.

Beginning with looking-time data, results revealed several significant effects for the high flexible infants, including a significant main effect of change status, *F*(1.54) = 6.049, *p* = 0.017, *partial eta^2^* = 0.101, and a significant main effect of set size, *F*(3165) = 4089, *p* = 0.008, partial *eta^2^* = 0.07 (Figure 7). The change status by set size interaction was not significant (*p* = 0.145). An inspection of the means suggests a clear signature of visual STM ability, including significantly longer looking to the change trials (i.e., change detection) and decreasing change preference with increasing set size. An examination of the simple main effects confirmed this impression: High flexible infants looked significantly longer at the change trials than to the no change trials for set sizes 1 and 2 (*p* = 0.019 and 0.015, respectively) but not for larger set sizes (all *p*s > 0.1). Results for the low reactive group revealed only a marginally significant main effect of set size, *F*(3123) = 2308, *p* = 0.080, and results for the high reactive group revealed no significant effects (Figure 7).

Turning now to the switch count analyses, results revealed only a significant main effect of set size which was present for all three phenotypes: High flexible infants, *F*(3162) = 96,970, *p* < 0.001, *partial eta^2^* = 0.642, low reactive infants, *F*(3123) = 46,314, *p* < 0.001, *partial eta^2^* = 0.530, and high reactive infants, *F*(3.12) = 8714, *p* = 0.002, *partial eta^2^* = 0.685. No other effects were significant, suggesting the effect driven by increases in visual complexity may simply overwhelm the substantially smaller change-detection effects we have seen in this measure (Figure 7).

### 3.3. Discussion

Results from our visual STM analyses revealed several interesting findings. First, consistent with previous research [58] measures of switch count and total looking during the test array did appear to capture important between-group differences in overall STM ability, and thus provided an excellent test of our hypothesis that attentional phenotype underpins general cognitive functioning and skill. Visual STM tasks are ideal for this purpose as they require a substantially different set of visual skills then a simple orienting task, and because STM ability is thought to influence myriad cognitive processes [68,69,70]. Two measures taken from this change detection task, switch count and total looking, revealed significant attention phenotype differences. Somewhat contrary to our initial expectations, high flexible infants demonstrated significantly longer look durations and significantly more switching overall than the low reactive infants. The high reactive infants were much more variable due to low sample size, though they produced longer look durations than the low reactive infants, and highly varied switch counts.

Results examining visual STM performance by phenotype group revealed a slightly more nuanced pattern of effects: High flexible infants looked significantly longer to change trials overall and this effect was strongest for set sizes 1 and 2. This pattern suggests sufficient visual STM capacity for arrays of up to two items, with diminishing performance as set size increased. In addition, though high flexible infants showed long look durations and high switch counts, both scores increased significantly with increasing set size, suggesting a skilled and flexible pattern of visual engagement—as visual complexity increased, switching and total looking increased to allow for greater encoding of the entire array. In contrast, results for both the low reactive and high reactive infants revealed no clear evidence of change detection even at the smallest set sizes. In addition, contrary to high reactive infants, look durations for the low reactive infants decreased with increasing set sizes. Though this effect was only marginal, this tendency coupled with the lack of evidence for change detection suggests low reactive infants may be struggling to deal with the effects of high visual competition, disengaging from the task before they are able to build a visual STM trace. Though switch counts do increase with increasing set size, the magnitude of this increase is insufficient for driving the longer look durations apparent for the high flexible infants at set sizes 3 and 4. Overall, only high flexible infants show evidence of change detection. This finding corroborates our initial impression that the high flexible attention phenotype may be more developmentally advantageous, and that attention phenotypes may help explain individual differences in cognitive performance.

## 4. General Discussion

The IOWA task is based on simple visual orienting responses, and previous work has demonstrated the importance of each of these skills for typical development [71]. However, in addition to measures of visual orienting, the IOWA task produces several scores that can be combined to help characterize attentional functioning across a variety of foundation attentional skills from spatial attention to saccade inhibition. Results from this longitudinal dataset reveal that by taking a multivariate approach to the measuring of attention (e.g., speed, accuracy, and spatial cueing effects) we can identify consistent behavioral patterns or phenotypes that predict distinct growth trajectories throughout the first year of life, and possibly beyond. Moreover, if individual differences in attention influence cognitive development more generally, then attentional phenotype may culminate in larger long-term differences. That is, if the identified attention phenotypes reflect individual differences in the underlying neurophysiological development, reactivity, and experience, then these differences should persist across a variety of visual cognitive tasks.

This paper presented results from two experiments, the first a longitudinal assessment of attentional development from 5 to 11 months of age, and the second a concurrent task designed to assess links between attention phenotype group discovered in Experiment 1, and cognitive functioning more generally. Specifically, using measures of visual orienting proficiency gleaned from the IOWA task [1], we have identified three distinct attentional styles or phenotypes that are stable from 5 to 11 months and possibly beyond. Importantly, these attentional phenotypes appear to be related cognitive development more generally, providing evidence that these phenotypic differences reflect individual differences in persistent, underlying patterns of behavior. This is an important finding and reveals that our phenotype clusters were apparent at all three ages and predicted qualitatively different patterns of development from 5 to 11 months. If these phenotypes merely reflected general maturation or noise, we would not expect the inclusion of phenotype in our model to improve model fits over-and-above our time only baseline model. This analysis provides strong support that all three phenotypes, including the high reactive phenotype, reflect persistent behavioral patterns that give way to significant and meaningful differences in growth trajectory.

Interestingly however, phenotype group significantly improved growth modeling for only four of the six IOWA attention scores. These scores were cue facilitation, or the amount of benefit incurred from a valid spatial precue, cue interference, or the amount of decrement incurred from an invalid spatial cue, task error, the rate of errors averaged across the two high inhibition conditions (invalid cue and double cue), and mean reaction time. In contrast, the time only baseline model provided best fits for both baseline error (mean error rates for valid, no cue and tone cue conditions) and cue competition (decrement incurred from double spatial cue relative to valid spatial cue), suggesting these scores mostly likely reflected general maturational trends. This is interesting, as the double cue condition was designed to elicit strong individual differences in more cortically-mediated visual orienting [44,61]. Given the attention cues in the double condition were presented simultaneously to the left and right of fixation, it is possible that the interhemispheric communication needed to detect simultaneity simply slowed the orienting response sufficiently in all groups. This cannot be the entire story, however, as error rates did increase for these conditions, suggesting that despite the simultaneous cues and subsequent visual competition, infants were still able to program and execute a saccade rapidly enough to produce the error.

Results from Experiment 2 demonstrate that these attentional phenotypes are not only stable in the context of a visual attention task, but also produce significant differences in performance on a visual STM task designed to assess differences in general cognitive development. Given the substantially different task parameters of the IOWA task and the visual STM task (e.g., speeded 100 ms cued trials vs. 3000 ms change detection trials), and given findings of distinct and stable growth trajectories of the unique phenotypes, it seems likely that the link between attentional phenotype and subsequent performance on our visual STM task are both mediated by the same underlying neurophysiology. That is, the same underlying neurophysiological state that contributes to the fast reaction times and strong spatial attention scores exhibited by the high flexible infants during the IOWA task, may also contribute to the increased scanning and look durations that facilitated their particularly good performance on the visual STM task. We speculate that rapid, accurate performance in the context of the IOWA task requires two things: Ample development of subcortical orienting mechanisms that support reflexive saccades (e.g., sufficient myelination, and increased synaptic development) [9,27] and sufficient cortical development to support saccade inhibition when visual competition is high (e.g., substantial development of frontal eye fields and parietal cortex) [6,40]. Thus, it seems that our high flexible infants are showing a more adaptable pattern of responding earlier in development. This conclusion is further supported by our concurrent visual STM task—only the high flexible infants demonstrated clear evidence of change detection. It remains to be seen if this flexible attention profile gives way to a persistent cognitive boost across childhood.

The foundational importance of these first months of visual learning cannot be overstated. For example, in our data, we have found that 5- to 11-month-old infants make approximately 1.79 saccades/s when viewing a naturalistic scene comprised of novel objects [72]. Based on this general scanning rate, infants may scan as many as 6000 objects each day. Even a small decline in saccadic rate to 1.5 saccades/s, could substantially reduce visual object exploration. Moreover, this visual exploration deficit compounded over six months could result in nearly 200,000 fewer scanned objects before the end of the first year of life. Thus, we believe even if attentional phenotypes do no mediate all cognitive development, they most certainly contribute to it through increased visual learning. Clearly high flexible infants demonstrate fast saccades (Experiment 1) and high saccade rates (Experiment 2). However, what about our remaining two phenotypes?

Low reactive infants in general demonstrate relatively low spatial attention, and relatively slow reaction times. As a result, it is not surprising that they have relatively low task error rates, as saccade programming occurs so slowly that the target is typically present before infant initiates the saccade. Although it may seem counterintuitive, low error rates that do not increase with subsequent development signify developmental immaturity, likely due to relatively slow processing speed. Consistent with this observation is the striking lack of improvement for spatial attention scores. The high reactive infants are a bit more puzzling. Though the prevalence rate is low (5.4% of our sample versus 39.6% for the low reactive, and 55% for the high flexible), removing them from the sample statistically weakens our latent profile analysis. Thus, it is possible that it characterizes infants at risk for neurobehavioral deficits. Behaviorally, these infants tend to make very fast eye movements and show strong spatial attention, however they appear to lack saccade inhibition, a key cortical ability that facilitates encoding and memory [13,14]. This manifests as very high error rates and very high cue interference scores that do not appear to improve with age. This phenotype bears some resemblance to patterns of visual behavior demonstrated by children diagnosed with attention deficit hyperactivity disorder (ADHD) [73]. Although purely speculative, the prevalence rate of ADHD in children is similar to that captured by our high reactive phenotype [74]. Thus, multivariate orienting measures may help to identify problematic attention functioning early in infancy when interventions are most successful.

All three attentional phenotypes produce qualitative differences in growth trajectory from 5 to 11 months that are readily explained through known neurophysiological developmental mechanisms. Thus, although evidence of distinct latent profiles alone is insufficient evidence for meaningful phenotypic differences, results from Experiments 1 and 2 provide strong support for their existence. First, these profiles produced stable and distinct growth trajectories for four attention scores that cannot be explained through development alone. Second, these profiles are behaviorally meaningful and theoretically grounded in well-known neurodevelopmental events including general improvements in neural connectivity and transduction [27,34,35], decreased neural noise [36], and maturation of cortical vision and saccade inhibition [2,12,26,44]. Lastly, these behavioral profiles are meaningful beyond simple visual orienting tasks, predicting performance on a separate visual STM task: High flexible infants showed strong evidence of change detection for set sizes 1 and 2, whereas neither low reactive nor high reactive phenotypes showed such evidence. It is currently unknown if underlying differences in neurophysiology influence both IOWA and visual STM performance directly, or if neurophysiology influences visual orienting patterns which in turn influence visual STM and presumably, general visuo-cognitive performance. One way to begin to address this question is to use neuroimaging and computational modeling to look for correlations between underlying neural state, and performance on both tasks. Another approach is to follow these infants longitudinally in an attempt to see if attention phenotype in infancy predicts group differences in general developmental outcomes. This work is currently underway.

## 5. Conclusions

Taken together, results presented here demonstrate the utility of simple attentional orienting tasks for assessing both attentional development, and cognitive development more generally. Infants tested in the IOWA task showed consistent patterns of orienting that clustered into three phenotype groups, each of which produced distinct growth trajectories from 5 to 11 months. Moreover, the importance of attention phenotypes may extend beyond simple orienting tasks, here producing significantly different patterns of performance on a separate visual short-term memory tasks. This suggests that phenotypic differences in attentional style may contribute to emerging differences in other foundational cognitive skills such as comparison, memory, and categorization. Thus, tasks such as the IOWA task and other visual orienting tasks hold much promise for use as early markers of risk and resilience.

## Figures and Tables

**Table 1:** 11-month-old latent profile analysis (LPA) model fits. Note: *n* = 111. The LMR test compares the current model to a model with k-1 profiles. LogLik = log likelihood; AIC = Akaike information criterion; BIC = Bayesian information criterion; LMR = Lo-Mendell Ruben likelihood ratio test.

Model	Profile Count (k)	logLik	AIC	BIC	Entropy	Smallest Profile (%)	LMR (*p*-Value)
m1	1	−31.19	87.38	119.53			
m2	2	16.44	4913	56.94	0.854	38.7/61.3	<0.000
m3	3	42.62	−32.24	38.24	0.892	5.4/55	0.306
m4	4	65.52	−64.04	24.11	0.909	5.4/49.6	0.358
m5	5	80.93	−80.87	27.94	0.896	4.5/46.9	0.223

**Table 2:** Attention score means and standard deviations by phenotype and age.

		High Flexible		High Reactive		Low Reactive	
		Mean	SD	Mean	SD	Mean	SD
5 mos	Cue Facilitation	0.356	(0.307)	0.449	(0.502)	0.248	(0.252)
	Cue Interference	0.068	(0.699)	0.618	(0.326)	0.175	(0.291)
	Cue Competition	0.135	(0.350)	−0.060	(0.410)	0.196	(0.168)
	Baseline Error	0.065	(0.070)	0.091	(0.053)	0.062	(0.066)
	Task Error	0.411	(0.157)	0.611	(0.152)	0.283	(0.201)
	Mean SRT	283.787	(77.912)	259.137	(89.947)	310.570	(69.786)
8 mos	Cue Facilitation	0.581	(0.321)	0.563	(0.187)	0.315	(0.365)
	Cue Interference	0.328	(0.865)	0.705	(0.599)	0.223	(0.308)
	Cue Competition	0.283	(0.208)	0.730	(0.782)	0.202	(0.239)
	Baseline Error	0.062	(0.067)	0.030	(0.026)	0.047	(0.058)
	Task Error	0.544	(0.118)	0.586	(0.143)	0.361	(0.213)
	Mean SRT	243.302	(59.068)	267.673	(110.289)	314.393	(106.944)
11 mos	Cue Facilitation	0.725	(0.297)	0.756	(0.256)	0.318	(0.160)
	Cue Interference	−0.013	(0.386)	1.725	(0.568)	0.160	(0.327)
	Cue Competition	0.328	(0.292)	0.267	(0.250)	0.254	(0.200)
	Baseline Error	0.066	(0.053)	0.059	(0.075)	0.042	(0.043)
	Task Error	0.593	(0.087)	0.641	(0.062)	0.321	(0.128)
	Mean SRT	204.490	(38.350)	283.817	(32.826)	310.912	(72.211)

**Table 3:** Linear mixed effects (LME) and conditional growth curve analysis for the high flexible and high reactive profiles compared to the low reactive profile.

	Cue Facilitation	Cue Interference	Cue Competition	Baseline Error	Task Error	Mean SRT
Age	0.029	−0.016	0.03	−0.01	0.01	0.677
	(−0.039)	(−0.078)	(−0.038)	(−0.008)	(−0.017)	(−8.752)
High Flexible	−0.055	0.016	−0.1	−0.004	0.037	13.147
	(−0.117)	(−0.235)	(−0.115)	(−0.024)	(−0.055)	(−26.936)
High Reactive	0.034	−0.309	−0.179	0.017	0.267 *	−63.875
	(−0.231)	(−0.516)	(−0.227)	(−0.047)	(−0.107)	(−52.898)
Age*High Flex.	0.155 **	−0.039	0.063	0.01	0.078 ***	−40.032 ***
	(−0.048)	(−0.098)	(−0.048)	(−0.01)	(−0.022)	(−10.835)
Age*High React.	0.13	0.601 **	0.104	−0.003	0.011	12.373
	(−0.097)	(−0.212)	(−0.097)	(−0.019)	(−0.043)	(−21.812)
Constant	0.238 *	0.219	0.164	0.070 ***	0.301 ***	309.477 ***
	(−0.097)	(−0.192)	(−0.094)	(−0.02)	(−0.045)	(−22.173)
Observations	240	224	240	240	240	240
logLik	−38.277	−186.163	−29.951	344.025	141.288	−1344.52
AIC	92.553	388.327	75.901	−672.051	−266.576	2705.04
BIC	120.398	415.62	103.746	−644.206	−238.731	2732.89

Note: * *p* < 0.05; ** *p* < 0.01; *** *p* < 0.001.

**Table 4:** Comparison of time-only baseline model (m1) versus model containing main effects and time interactions for the latent profiles (m2).

Attention Score	Model	df	AIC	BIC	logLik	Test	L. Ratio	*p*-Value
Cue Facilitation	m1	4	139.56	153.48	−65.78			
	m2	8	92.55	120.40	−38.28	1 vs. 2	55.01	<0.001
Cue Interference	m1	4	426.39	440.04	−209.20			
	m2	8	388.33	415.62	−186.16	1 vs. 2	46.07	<0.001
Task Error	m1	4	−181.52	−167.60	94.76			
	m2	8	−266.58	−238.73	141.29	1 vs. 2	93.06	<0.001
Mean SRT	m1	4	2762.47	2776.40	−1377.24			
	m2	8	2705.04	2732.89	−1344.52	1 vs. 2	65.43	<0.001
Cue Competition	m1	4	71.47	85.39	−31.73			
	m2	8	75.90	103.75	−29.95	1 vs. 2	3.57	0.468
Baseline Error	m1	4	−674.80	−660.88	341.40			
	m2	8	−672.05	−644.21	344.03	1 vs. 2	5.25	0.263
